# Diet Drives Gut Bacterial Diversity of Wild and Semi-Captive Common Cranes (*Grus grus*)

**DOI:** 10.3390/ani14111566

**Published:** 2024-05-25

**Authors:** Hong Wu, Nan Wu, Xinchen Liu, Lei Zhang, Dapeng Zhao

**Affiliations:** 1 Tianjin Key Laboratory of Conservation and Utilization of Animal Diversity, College of Life Sciences, Tianjin Normal University, 393 West Binshui Road, Xiqing District, Tianjin 300387, China; skywuhong@tjnu.edu.cn (H.W.);; 2 Beijing Wildlife Park, Daxing District, Beijing 102602, China

**Keywords:** high-throughput sequencing, fecal flora, *Grus grus*, diet

## Abstract

**Simple Summary:**

The fecal microbiome of common cranes (*Grus grus*) in Tianjin Tuanbo Bird Natural Reserve (wild group) and Beijing Wildlife Park (semi-captive group) in China were analyzed and compared in this study, and the results showed that Firmicutes and Proteobacteria are the dominant phyla. The Sobs and Chao1 indexes revealed that the richness of gut microbiota in the semi-captive group was higher than in the wild group. Among the analysis of the relationship between plants and intestinal bacteria, *Zea mays*, *Glycine max*, and *Phragmites australia* had a significant correlation with the intestinal bacteria of *G. grus*. Beta diversity data highlighted the significant differences between the two groups and that the gut microbial taxa of the *G. grus* were associated with the main functions to adapt the dietary compositions. Taken together, these data will help us evaluate the influence of diet on animal microbiomes and improve our ability to enhance the conservation of this species.

**Abstract:**

The gut microbiota of wild animals can regulate host physical health to adapt to the environment. High-throughput sequencing from fecal samples was used to analyze the gut microbiota communities in common cranes (*Grus grus*) without harming them. Herein, we compared the fecal microbiome of fifteen *G. grus* in Tianjin Tuanbo Bird Natural Reserve (wild group) and six *G. grus* sampled from Beijing Wildlife Park (semi-captive group) in China, using 16S amplicon sequencing and bioinformatic analysis. The results showed that microbiota diversity and composition varied in different groups, suggesting that the gut microbiota was interactively influenced by diet and the environment. A total of 38 phyla and 776 genera were analyzed in this study. The dominant phyla of the *G. grus* were Firmicutes and Proteobacteria. Meanwhile, the microbiota richness of the semi-captive group was higher than the wild group. Data on beta diversity highlighted significant differences based on different dietary compositions. *Zea mays*, *Glycine max*, and *Phragmites australia* showed a significant correlation with intestinal bacteria of *G. grus*. This study provides a comprehensive analysis of diet and microbiomes in semi-captive and wild *G. grus* living in different environments, thus helping us to evaluate the influence on animal microbiomes and improve conservation efforts for this species.

## 1. Introduction

Recent studies have demonstrated that the bacterial communities of the gut are closely associated with host health [1]. Many factors including host genetics, living conditions, diet, and stress affect the diversity and composition of the gut microbiota. Among these influences, living conditions and diet have significant effects on bacterial communities. Since food is the main source of energy [1,2], the relationship between the gut microbiota and nutritional intake is critical for the survival of wild animals [3]. It is important to evaluate the influences of diet and living conditions on the relative abundance of gut microbiota and consequences for animal health [4]. In studying the effects of the living environment on the host microbiome, individual zoo animals under managed care can be particularly useful because these environments are relatively simple, with fewer variables compared to the wild [5]. Like other vertebrates, Firmicutes and Proteobacteria dominate the intestinal microbes of birds, which differs significantly from mammals and insects [6]. At present, the research on the intestinal microbiota of wild animals mainly focuses on mammals, there are relatively limited studies on the intestinal microbiota of birds. The birds, with their complex life history and strong flying ability, often have rich species diversity and genetic diversity. The composition and function of intestinal microorganisms in wild birds—especially migratory birds—remains to be further studied. 

The common crane (*Grus grus*) belongs to the crane genus, listed as an LC (Least Concerned) species by the International Union for Conservation of Nature (IUCN 2020) [7]. *G. grus* is listed as a national second-class protected species in China. As large migratory wading birds that widely spread, *G. grus* starts the migration from their breeding places every autumn to the wintering habitat including Tianjin city of China, then return the following spring [8]. Like many migratory birds [9,10], the survival of the *G. grus* is affected by human activities and environmental changes. 

Previous studies have shown that many migrating birds, such as hooded cranes (*Grus monacha*) [11], greylag geese (*Anser anser*) [12], and swan geese (*Anser cygnoides*) [13], are able to adapt to the local environment in response to seasonal and diet variations by regulating the gut microbiota [14,15]. In addition to being affected by the living altitude [16], the diversity and composition of gut microbiome are also driven by host species and dietary nutrients [17]. Diet is a key factor affecting microbial diversity for animals, with the matching of the microbiome with diet being one potentially important mechanism to overcome changes throughout the whole life cycle [18]. The dietary change could cause the loss or extinction of certain intestinal microbes in response to the variations in living environment [19]. The common crane is an omnivore and feeds on plants including roots, rhizomes, tubers, and seeds. During the breeding season, they also eat insects, especially dragonflies, crabs, and spiders. With the rapid development of high-throughput sequencing technology, isolation of the microbial composition from fecal samples has become a useful method to identify intestinal microbial community composition [20,21,22,23]. Some mammalian studies have shown that the diversity of gut microbial composition is influenced by habitat quality [24]. However, for wild animals, the impact of living and dietary conditions on the composition of gut microbes outside the laboratory remains a complex issue [25,26]. 

The aim of this study was to characterize and compare the gut microbiota of semi-captive *G. grus* living in Beijing wildlife park, and wild birds from Tianjin Tuanbo Bird Natural Reserve, in order to explore the relationship between diet and intestinal microbial diversity. The intestinal flora of wild birds is affected by external factors, such as diet and living environment, but how the composition of food affects the structure of the gut flora and how birds regulate the gut flora to adapt to changes in the external environment need to be further studied. The results are helpful to analyze the adaptation mechanism of animal intestinal flora to the environment and provide a reference for the wild protection of *G. grus*. 

## 2. Materials and Methods

### 2.1. Fecal Sample Collection and DNA Extraction

The sample collection complied with the current laws of China and followed the applicable international and national guidelines. Fecal samples of common cranes were collected without direct contact. The common cranes in the wild from the Tianjin Tuanbo Bird Natural Reserve mainly foraged freely, while the food for semi-captive group from Beijing Wildlife Park was provided by zookeepers (Supplemental Table S1). 

In our study, a total of six fresh fecal samples of semi-captive *G. grus* living in the Beijing Wildlife Park (BYG group) and fifteen samples from the Tianjin Tuanbo Bird Natural Reserve (TBG group) were collected by the non-invasive sampling method. For collecting stool from wild animals, the identity and age of the samples were unknown. However, these parameters were indeed known from the semi-wild birds in the park. The sample collections were conducted shortly after the *G. grus* was observed. After placing them into sterile fecal collection tubes, all samples were temporarily stored at −20 °C and later transferred to −80 °C for later DNA extraction and microhistological analysis. 

### 2.2. DNA Extraction and Sequencing

Total DNA from each fecal sample was extracted by QIAamp DNA Stool Mini Kit (QIAGEN, Hilden, Germany). The quality of DNA was measured using nanodrop2000 and the integrity was verified by 2.0% (*w*/*v*) agarose gel electrophoresis. The pair of universal bacterial barcoded primers (515F: 5′-GTGCCAGCMGCCGCGG-3′and 907R: 5′-CCGTCAATTCMTTTRAGTTT-3′) were used to amplify the 16S rRNA V3–V4 regions of all the microorganisms in the fecal samples by polymerase chain reaction (PCR). The PCR amplification was carried out in triplicate 20 μL reactions with 10 ng sample DNA, 10 μL Taq PCR Master Mix (2×), 0.8 μL of each primer (5 μM), and certified water to reach the necessary 20 µL volume. The PCR conditions were as follows: 98 °C for 3 min (initial denaturing), 30 cycles of 95 °C for 30 s (denaturing), 55 °C for 30 s (annealing), 72 °C for 45 s (extension), and finally 72 °C for 10 min. The DNA library was sequenced on the Illumina NovaSeq PE250 platform (Illumina, San Diego, CA, USA) at LC-Bio Technology Co., Ltd. (Hangzhou, China). 

### 2.3. Bioinformatics Analysis

The raw fastq data of all the samples were demultiplexed and low-quality base pairs were removed from the paired-end reads. The samples were sequenced to a depth of 10,000 minimal reads and Operational taxonomic units (OTUs) taxonomy with 97% sequence similarity was assigned using the de novo UClust method. These representative sequences of OTU were assigned against the Silva 128 database (https://www.arb-silva.de, accessed on 10 April 2023) [27] and could be used in downstream statistical analyses. The alpha diversity indices including Shannon, Simpsoneven, Sobs, and Chao1 were calculated in this study using mothur (version v.1.30.1, https://www.mothur.org/, accessed on 10 April 2023) and the differences of alpha diversity were analyzed based on a Wilcoxon’s test. 

The beta diversity of the gut microbiota was calculated based on weighted and unweighted UniFrac distances, and principal coordinates analysis (PCoA) plots were created to visualize the differences between the two groups. The effect of different breeding ecological environment was tested using ADONIS, through a multivariate analysis of variance with 999 permutations, in order to find the difference between two groups [28]. The hierarchical clustering heatmap was carried out at OTU-level taxa to visualize the relationship between fecal samples from two groups by MicrobiomeAnalyst [29]. A one-way analysis of variance (ANOVA) and LefSe (Linear discriminant analysis Effect Size) were both used to determine the differences in the abundance of the gut microbiota.

### 2.4. Diet and Microhistological Analysis

Plants consumed by the *G. grus* were identified by microhistological analysis. Plant leaves and seeds were collected from the Tianjin Tuanbo Bird Natural Reserve and Beijing Wildlife Park. Availability of plant species was based on long-term vegetation monitoring plots. To prepare fecal pellet samples of common cranes for microhistological analysis using the concentrated nitric acid method [30]. 

The fecal samples were placed in an oven until the weight was constant, and all the dried material were screened using a net screen of 200 mesh. Then, the materials were digested in 4% (*v*/*v*) sodium hypochlorite. In order to stain epidermal fragments, plant samples were first rinsed under running water, and then incubated in a 1% (*v*/*v*) aqueous solution of gentian violet for 60 s. They were then washed twice with water prior to a final incubation in a 70% (*v*/*v*) ethanol solution. The material was transferred to a microscope slide and photographed at 10× magnification to observe the plant cell morphology. These images were taken on a Nikon microscope and provided the basis to identify the plant fragments in the fecal samples of *G. grus*. 

After mounting on a microscopic slide, the samples were moved systematically at 10× magnification until 100 fragments were identified per slide. The species of plant were identified based on the established plant cell morphological atlas database. Although there was some overlap in the epidermal features, several key plant species in the fecal samples of *G. grus* were identified by distinct diagnostic features at the family/genus level. The recorded data were used to analyze the composition of plants in feces, including the frequency (F) of each plant (F represents the frequency of each plant in 100 fields of view), average density (D)(D = −ln (1 − F/100)), and relative density (RD)(RD = (D/∑D) × 100%) [30]). A Mantel test was used in this study to evaluate the correlation between food composition and intestinal bacteria.

## 3. Results

### 3.1. Microbial Community Diversity and Alpha Diversity

A total of 1,674,611 reads and 2685 OTUs were obtained from 21 fecal samples by 16S rRNA amplicon sequencing, and the average sequence length was 450 bp (Supplemental Table S2). The rarefaction curves tended toward saturation in two groups, and the data quality was reliable, indicating that the sequencing data volume of these samples was sufficient to reflect the gut microbial information (Supplemental Figure S1). 

OTU clustering was performed for non-repeating sequences according to 97% similarity, and the observed numbers of OTUs could be used as a qualitative measure of community richness to indicate the alpha diversity. By this metric, there were 991 OTUs shown to be shared by both the BYG group and TBG group under different living conditions. The BYG group harbored higher numbers of OTUs compared to the TBG group at the same sequencing depth (Supplemental Figure S2). 

**Figure 1:**
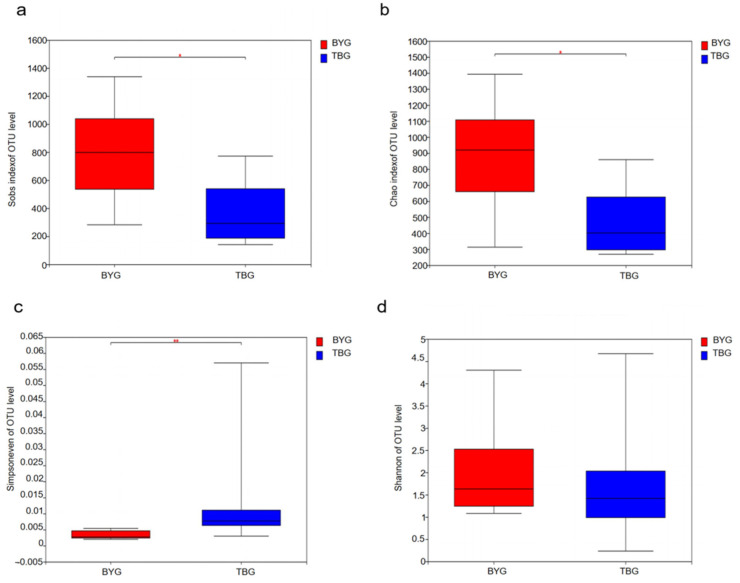
The alpha diversity between the BYG group and TBG group: (**a**) Sobs diversity index of OTU level, (**b**) Chao1 index of OTU level, (**c**) Simpsoneven index of OTU level, and (**d**) Shannon index of OTU level. *p* values were calculated using Wilcoxon rank-sum tests. * *p* < 0.05, ** *p* < 0.01.

In our study, a series of statistical indexes was analyzed through alpha diversity to estimate the species abundance and diversity of microbial communities (Table 1). The Wilcoxon rank-sum tests, the Sobs diversity index, Simpsoneven index, and the Chao1 index showed high variation between the two groups. The Sobs and Chao1 indexes reflected the richness while the Simpsoneven index reflected the evenness of gut microbiota in both the BYG and TBG groups. The richness of the microbial communities in the BYG group was higher than in TBG group, and statistically significant differences were found between the two groups (*p* < 0.05) using the Sobs and Chao1 metrics (Figure 1a,b). The community evenness estimated by the Simpsoneven index was significantly different (*p* < 0.01) (Figure 1c), while the Shannon indices were not significantly different between the two groups (*p* > 0.05) (Figure 1d).

### 3.2. Taxonomic Comparisons of Gut Microbiota between Two Groups

**Figure 2:**
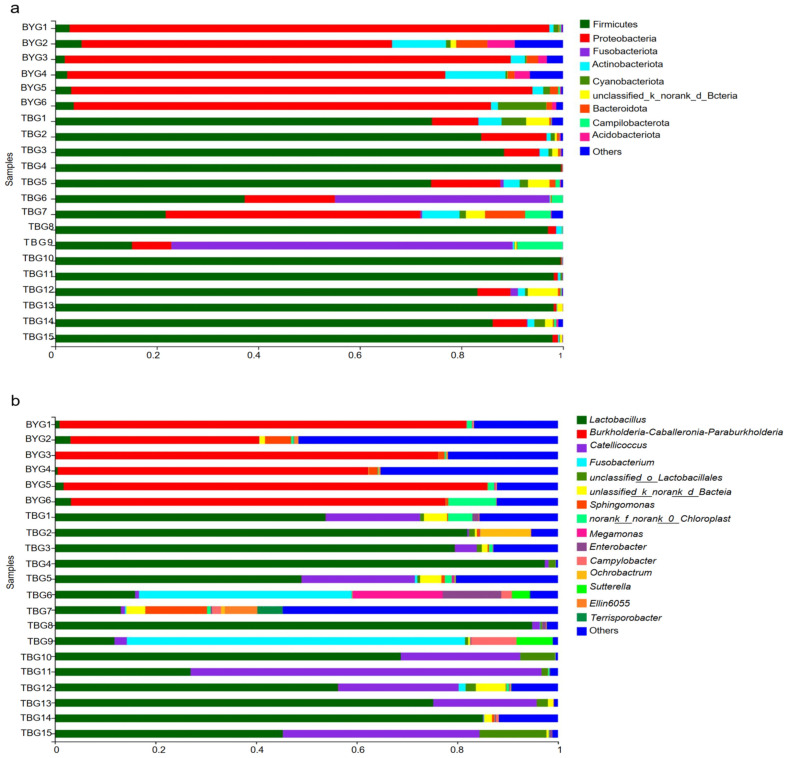
Microbial structure of all fecal samples at the phylum (**a**) and genus levels (**b**). Stacked columns for the means of the individual samples from the two groups, indicating the relative abundance as a percentage of the total bacterial sequences per sample. The taxa that have relative abundance of less than 1% were combined and are referred to as “others”.

**Figure 3:**
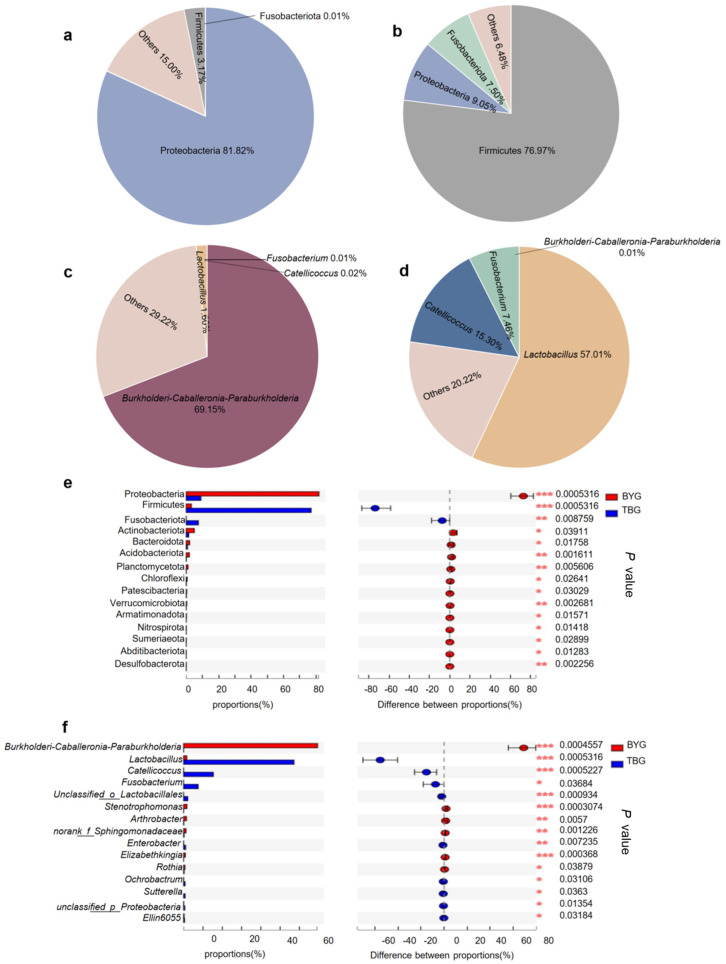
The relative abundance (%) of bacterial phyla/genus in the gray crane gut microbiome samples and differentially abundant in the gut microbiome between the two groups. The relative abundance (%) of bacterial phyla in the BYG group (**a**) and TBG group (**b**); the relative abundance (%) of bacterial genus in BYG group (**c**) and TBG group (**d**); the phyla and genus with a median relative abundance of less than 1% were collapsed into the category “Other”; and differential analysis of the dominant bacterial phyla (**e**) and genera (**f**) between the BYG group and TBG group based on the Wilcoxon rank-sum test. * *p* < 0.05, ** *p* < 0.01, and *** *p* < 0.001.

The OTUs were classified into 38 phyla, 104 classes, 245 orders, 405 families, and 776 genera in total. The gut microbiota of the captive gray cranes in the BYG group was presented by 37 bacterial phyla and 663 microbial genera, while there were 29 bacterial phyla and 594 microbial genera in the wild gray cranes in the TBG group. The bacterial read profiling of 16S rRNA sequencing at phylum level (Figure 2a) and genus level (Figure 2b) of each sample from two groups were analyzed in this study. The dominant phyla of the BYG group were Proteobacteria (81.82%), Actinobacteria (4.94%), and Firmicutes (3.17%). However, Firmicutes (76.97%), Proteobacteria (9.05%), Fusobacteria (7.50%), and Actinobacteria (1.52%) were the dominant phyla in TBG group (Figure 3a,b). The high-throughput sequencing approach identified Firmicutes, Actinobacteria, and Proteobacteria, as the most abundant phyla in all the fecal samples from *G. grus*, although the proportion of Proteobacteria and Firmicutes differed substantially. At the genus level, the most abundant genera in BYG group were *Burkholderia–Caballeronia–Paraburkholderia* (69.15%) and *Lactobacillus* (10.06%). Compared with the BYG group, the samples in TBG group were more enriched in *Lactobacillus* (57.01%) and *Catellicoccus* (10.06%) (Figure 3c,d). Further comparisons of microbial composition between the two groups were performed at the family level and class level based on 16S sequencing results (Supplemental Figures S3 and S4). 

**Figure 4:**
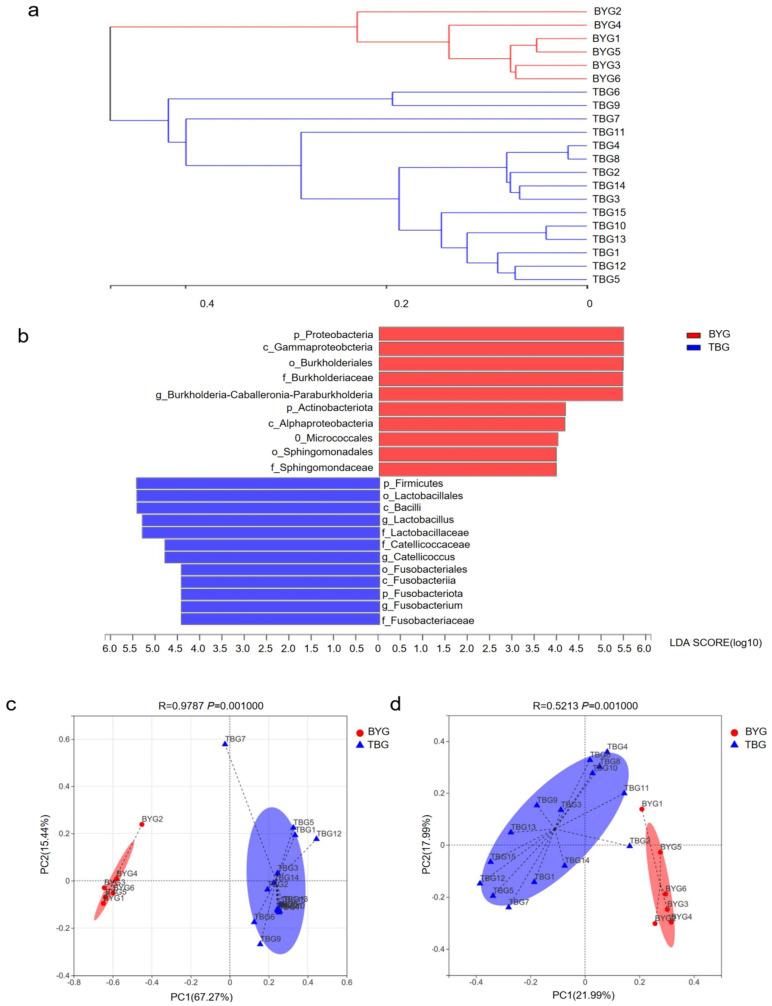
Hierarchical clustering analysis, LEfSe analysis of the gut microbiota, and PCoA plot analyses. (**a**) Hierarchical clustering analysis of the gut microbiota in all the fecal samples at the genus level based on Bray–Curtis; (**b**) LEfSe analysis of two groups, the length of the bar represents the contribution of the different species (LDA Score). The figure shows the species with significant differences between the different groups under the condition that the LDA Score is 4. (**c**) PCoA plots based on weighted UniFrac distances of gut microbiome. (**d**) PCoA plots based on unweighted UniFrac distances. Each point represents a unique gut microbiome sample.

The Wilcoxon rank-sum analysis showed significant differences in many of the phyla, mainly in the Proteobacteria (*p* = 0.0005) and Firmicutes (*p* = 0.0005). At the genus level, significant decreases were seen in *Burkholderia–Caballeronia–Paraburkholderia* (*p* = 0.0004) in the TBG group. *Lactobacillus* and *Catellicoccus* in the TBG group displayed opposite results compared to samples from the BYG group, suggesting that they might be tightly linked to the external environment and dietary habits (Figure 3e,f). Hierarchical clustering analysis of the gut microbiotas in all fecal samples at the genus level using Bray–Curtis showed that the two different groups formed distinct clusters, indicating that there were clear differences between the two groups (Figure 4a). Next, linear discriminant analysis (LDA) and effect size (LEfSe) were used to detect the differences in relative abundance in the bacterial taxon. The data revealed that the differences were most significant from phyla to genus levels (Figure 4b). Results from the LEfSe analysis determined that Firmicutes, which make up >55% of gut microbiome communities in *G. grus*, had higher relative abundances in the TBG group, while Proteobacteria (composed >29% of gut microbiome communities) were more abundant in the BYG group (*p* < 0.05 for all). LEfSe analyses identified 22 bacterial taxa that explained these differences, including 10 and 12 taxa (LDA = 4.0) with discrepancies in relative abundance in the BYG group and TBG group, respectively.

### 3.3. Beta Diversity of G. grus from Different Groups

Principal coordinate analysis (PCoA) was performed based on weighted UniFrac (Figure 4c) and unweighted UniFrac (Figure 4d) distances to visualize the beta diversity of the bacterial communities between the two groups. Gut microbial analysis revealed the strong site-dependent differences indicated by PCoA analysis. Using weighted UniFrac distance, bacterial communities of BYG group were separated from the TBG group along the principal coordinate axis 1 (PC1) with the largest degree of variation (67.27%). The data showed a significant separation (R = 0.9787, *p* = 0.001) (Figure 4c). The majority of samples from both the BYG and TBG groups were highly aggregated, bacterial communities of BYG group were separated from those of BTG group along main axis 1 (PC1) using unweighted UniFrac distances (R = 0.5213, *p* = 0.001), with the greatest degree of variation (21.99%) (Figure 4d). The weighted UniFrac analysis provided a much stronger clustering by population than the unweighted UniFrac, indicating that the clustering was likely driven by shifts in the ratios of dominant members of the microbiota. 

The similarity between groups was analyzed (ANOSIM/Adonis) to test the significance of gut microbial community composition differences between fecal samples from two groups. Analysis of similarities indices measured based on the unweighted UniFrac distance of all taxa metadata showed that the gut microbiota differed significantly from the different living environments (R^2^ = 0.5213, *p* = 0.001), and the ANOSIM results also revealed significant differences in bacterial communities between the two groups using weighted UniFrac distance (R^2^ = 0.9787, *p* = 0.001) (Supplemental Figure S5). These results were further consistent with the result of the beta PCoA analysis. Thus, these data indicated that gut microbiota composition differed significantly between the two groups.

### 3.4. Pathway Analysis between Different Groups

**Figure 5:**
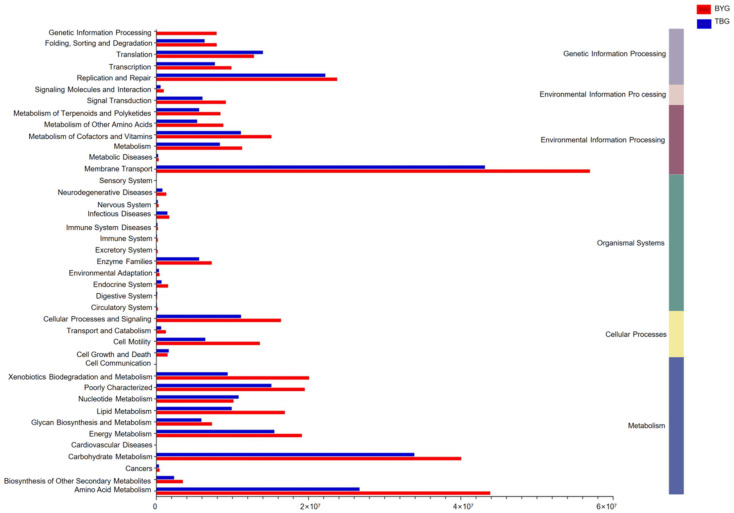
PICRUSt prediction of the functional abundance column diagram in pathway level 1/level 2.

Based on the results of predicted function, the genes related to various KEGG pathways were characterized by PICRUSt and a relative functional abundance column diagram in pathway level 1/level 2 was generated (Figure 5). 

At pathway level 1, all six categories of functional information including metabolism, organismal systems, and environmental information processing were analyzed and the differences in the abundances of genes related to various COG (level 2) and KEGG (level 2) categories between the two groups were investigated. At pathway level 2, membrane transport, and replication and repair were the main functions of the gut microbiome. The super pathway of heme biosynthesis from glutamate, aerobic respiration I (cytochrome c), and L−leucine degradation I of BYG group was significantly higher than the TBG group (*p* < 0.0001).

### 3.5. Microstructure Analysis and the Food Comparison 

The results of the microstructure analysis showed that the feces of wild *G. grus* in Tianjin were made up by nine species of plants belonging to nine genera and seven families (the full range of plant species in the wetland is described in Supplemental Table S3, and the microscopic structures of plant cell could be seen in Supplemental Figure S6). According to the microscopic results, the main proportion of the plants consumed in the TBG group were *Triticum aestivum* (50.05%), *Potentilla chinensis* (9.89%), *Zea mays* (8.77%), *Potentilla chinensis* (6.34%), *Glycine max* (5.78%), *Phragmites australis* (4.66%), and other unknown herbivorous foods (7.28%). Crops accounted for 69.21% of the herbivorous foods (Table 2). 

A total of six families, seven genera, and seven species of plants were found in feces of BYG group (the microscopic structures of plant cell is described in Supplemental Figure S7). The proportion of plants were mainly *Zea mays* (24.28%), *Glycine max* (21.22%), *Phragmites australis* (20.90%), *Capsella bursa* (10.93%), *Polygonum criopolitanum* (6.75%), *Acorus tatarinowii* (2.41%), and other unknown phytophagous foods (3.38%). Among them, crops accounted for 48.4% (Table 2). The cranes in the Beijing Wildlife Park are kept in a free-range state, but zookeepers regularly provide additional food, such as fresh fish. 

The Mantel test analysis, a non-parametric statistical method, showed that the main foods in the diet of *G. grus* have significant correlations with the gut microbiome. Among the common foods, *Zea mays*, *Glycine max*, and *Phragmites australia* had a significant correlation with intestinal bacteria, while *Potentilla amurensis* had no significant correlation (Table 3). Thus, the Mantel test based on OTU level using the Bray–Curtis matrix showed the preliminary evidence of a link between food and gut bacteria.

## 4. Discussion

The 16S rRNA high-throughput sequencing technology and noninvasive means of collecting fecal samples to study gut microbial have become important research tools for the protection of endangered wildlife. Various factors can affect the composition and diversity of gut microbiota, including food nutrition, and the living environment related with the type and quantity of food [31]. 

In this study, a total of 38 phyla and 776 genera were analyzed in all the fecal samples from *G. grus*. Among them, the population of the BYG group harbored higher numbers of OTUs than the TBG group at the same sequencing depth. More than half of all OTUs including *Lactobacillus*, *Catellicoccus*, *Fusobacterium*, and others were shared by two groups, and unique OTUs belonging to the BYG group were higher than the TBG group. Based on previous reports, the decrease in diversity was associated with a number of diseases [32,33,34]. For example, the content of pathogenic bacteria such as Fusobacteria increased in the TBG group, which increases their risk of acquiring diseases. This result is consistent with previous conclusions that diet affects the diversity and function of the host gut microbiome [35,36,37]. Alpha diversity, including community richness (Chao1 and ACE), community diversity (Shannon, Simpsoneven), were measured as the most common indicator for assessing gut microbiota. The higher the Simpsoneven and Shannon index value, the higher the community diversity. Alpha diversity was considered to be highly related to the occurrence and development of many diseases. The richness and evenness of microbial communities in the BYG group was significantly higher than in the TBG group (*p* < 0.05) using the Sobs and Chao1 metrics. Consistent with previous studies, our data showed that wild gray cranes from BYG have a higher alpha diversity than the *G. grus* from TBG [38]. *G. grus* from the BYG group have more diverse dietary sources including fish and plants in their living environment, thereby illustrating that high microbial richness is conducive to the survival of this species. The main food source for the TBG group is the plants in the Tianjin Tuanbo Bird Natural Reserve (Supplemental Table S3) with barely any intake of meat or fish. Therefore, higher alpha diversity of the gut microbiota probably benefits *G. grus* adaptation to food variety in different habitats, and the intake of fish or meat is conducive to improving the richness of the gut microbiota.

The dominant phyla of the *G. grus* intestinal bacteria were Firmicutes and Proteobacteria (Supplemental Figure S8), and this result is consistent with previous studies on other birds. Firmicutes contribute to the degradation of complex carbohydrates into volatile fatty acids, such as fiber and cellulose, and thus facilitating animal growth and survival in the wild [39]. The relative abundance of Firmicutes in the TBG group was significantly higher than in the BYG group, the differences observed in the intestine of *G. grus* may be related to the main plant source. *Triticum aestivum*, *Potentilla chinensis*, and *Zea mays* were the main food sources for *G. grus* from the TBG group during the wintering period. High levels of Firmicutes could contribute to the metabolism of the plant-based diet (cereal, vegetables, and fruit) [40], which is consistent with the results of this study, which show that the food in the TBG group contains more fiber compared to food in the BYG group. Butyrogenic bacteria, most of which belong to Firmicutes, can enhance fiber fermentation in *G. grus* to obtain energy from their plant-based diet [41,42].

At the genus level (Supplemental Figure S8), the abundance of *Burkholderia–Caballeronia–Paraburkholderia* was significantly higher in the BYG group. *Burkholderia* genomes have been reported to encode natural products with potential therapeutic relevance, such as in rice [43,44]. The TBG group, the abundance of *Lactobacillus* was much higher than that of the BYG group. Lactobacillus is best known for its use as a probiotic [45], participating in the maintenance of immunologic equilibrium in the gastrointestinal tract by direct interaction with immune cells. Further, the high abundance of *Lactobacillus* can help *G. grus* to quickly adapt to changes in different environments, but the nutritional interaction between gut microbiome and *G. grus* needs to be explored further. Our result was consistent with the hypothesis that varied diet indeed shapes the gut microbiota. 

The UniFrac method based on weighted/unweighted metrics developed by Lozupone and Knight focused on the differences and phylogenetic relationships between different groups [46]. In our study, the majority of gut microbiotas were conserved and clustered together under both weighted and unweighted UniFrac metrics among different breeding conditions, indicating that each fecal sample harbors different microbial communities. The differences in diet with relative protein, fat, and fiber content may be the main cause of the observed changes in the composition of the gut microbiota [3]. The diet structure remains stable in a semi-captive state, however, in order to ensure survival in the wild, *G. grus* have adapted to the environment not only through migration behavior but also convergent evolution in their gut microbiota. Findings in the present study demonstrate that gut microbiota respond to dietary variations across different living states, which allows *G. grus* to better utilize plant resources during overwintering periods for survival. The understanding of diet–gut microbiota interactions will improve our understanding of how *G. grus* adapt to environmental changes. PICRUSt was used to analyze the microbial functions of *G. grus*, and gut microbial taxa was associated with main pathways such as metabolism, organismal systems, and environmental information processing. These data facilitate our understanding of the relationship among the gut microbial taxa, diet, and the environment to gain better insight into wild animal health by comparing the composition of gut flora in different living environments. The relative abundance of Firmicutes in the TBG group was significantly higher than in the BYG group, which was related to plant sources. 

In summary, we hypothesized that differences in diet and breeding ecological conditions have great effect on gut microbiota of *G. grus*. In general, animals carry more diverse microbes when they have a wider variety of foods. The hypothesis was supposed by our data, which showed that high abundance of protein (fish/meat) allows *G. grus* to build a more diverse gut microbial system. Furthermore, the results on beta diversity suggested that individuals that are more similar in dietary composition share a similar gut microbiota in terms of evolution. This conclusion is consistent with previous studies focused on the effects of feeding habitats on animal gut microbiota. The residual plant samples in feces were subjected to macro-monitoring analysis, establishing a correlation between their proportion and the intestinal flora. Our findings revealed a significant association between intestinal microbes and certain plants including *Zea mays*, *Glycine max*, and *Phragmites australia*, and more in-depth metagenomic analysis is needed to explain the mechanism.

## 5. Conclusions

Our study focused on the analysis of the gut microbiota of wild and semi-captive *G. grus*. We reported the relationship between diet, ecological breeding conditions, and gut microbiota in wild animals using high-throughput sequencing. The abundance and diversity of the gut microbiome can be changed in response to changes in diet and breeding environment. However, strictly controlled experiments are required to explore this relationship and future research needs to determine how diet diversity is associated with gut microbial diversity. The results in this study show that Firmicutes and Proteobacteria are the dominant phyla of *G. grus* intestinal bacteria. The Sobs and Chao1 indexes revealed that the richness of the gut microbiota in the semi-captive BYG group was higher than in the wild TBG group, because the BYG group had a more diverse dietary source including fish/meat and plants. Among the analysis of relationship between plant and intestinal bacteria, *Zea mays*, *Glycine max*, and *Phragmites australia* had a significant correlation with intestinal bacteria of *G. grus*. Beta diversity data highlighted the significant differences between the two groups and that the gut microbial taxa of the *G. grus* were associated with the main functions to adapt the dietary composition. Taken together, these data will help us evaluate the influence of diet on animal microbiomes and improve our ability to enhance the conservation of this species.

## Figures and Tables

**Table 1:** Alpha diversity was used to estimate the species abundance and diversity of microbial communities.

Sample	Sobs	Simpsoneven	Chao1	Shannon	Ace	Coverage
BYG1	284	0.005385	316.2826	1.174740	324.2621	0.999194
BYG2	1333	0.005165	1375.0620	4.296252	1374.3710	0.998564
BYG3	863	0.001994	950.4793	1.786709	845.7505	0.997861
BYG4	1104	0.002353	1191.3440	2.767240	1168.6470	0.998095
BYG5	461	0.003055	580.1563	1.076843	560.8193	0.998183
BYG6	739	0.002397	939.1724	1.465221	926.8143	0.996835
TBG1	621	0.005376	714.4304	2.358204	707.4409	0.998212
TBG2	546	0.003534	705.0723	1.414751	687.7625	0.997612
TBG3	280	0.006499	323.3636	1.497142	323.6567	0.999209
TBG4	139	0.007739	229.2308	0.233573	324.2930	0.998989
TBG5	528	0.012882	602.6522	2.817375	609.5538	0.998505
TGB6	173	0.024830	285.8571	2.007119	394.1489	0.998828
TBG7	766	0.056894	849.7391	4.664402	839.9976	0.998418
TBG8	196	0.006157	328.0000	0.529410	410.3215	0.998711
TBG9	190	0.011155	293.0526	1.309319	422.3113	0.998696
TBG10	174	0.010918	384.0370	0.919415	634.3366	0.998432
TBG11	295	0.006109	427.2222	0.892827	524.5832	0.998242
TBG12	428	0.008311	533.0196	2.051407	523.7572	0.998476
TBG13	268	0.007102	351.1429	1.047081	443.1174	0.998579
TBG14	544	0.003021	636.6389	1.465143	642.9121	0.998300
TBG15	350	0.008212	467.2979	1.368138	511.8669	0.997817

**Table 2:** Comparison of diet composition between the BYG and TBG groups based on the relative frequency of each plant in fecal samples.

Family	Species	TBG			BYG		
		F (Times)	D (%)	RD (%)	F (Times)	D (%)	RD (%)
Gramineae	*Triticum aestivum*	268	50.13	50.05			
	*Zea mays*	47	8.77	8.76	151	24.31	20.92
	*Phragmites australis*	25	4.67	4.66	130	20.92	18.01
Malvaceae	*Abutilontheophrasti*	24	4.48	4.47			
Cyperaceae	*Carexbreviculmis*	34	6.35	6.34			
Rosaceae	*Potentilla chinensis*	53	9.89	9.88	63	26.24	22.58
Leguminous	*Glycine max*	31	5.79	5.78	132	21.24	18.28
Ranunculaceae	*Ranunculus japonicus*	7	1.31	1.30			
Composite	*Artemisia capillaris*	8	1.49	1.49			
Polygonaceae	*Polygonum criopolitanum*				42	6.75	5.81
Cruciferae	*Capsella bursa*				68	10.93	9.41
Araceae	*Acorus tatarinowii*				15	2.41	2.08
	Others	39	7.28	7.27	21	3.38	2.91

**Table 3:** Plants related to the composition of the intestinal bacteria of *G. grus* according to the Mantel test.

Plant	R	*p*-Value
*Zea mays*	0.43470	0.003
*Glycine max*	0.53895	0.006
*Phragmites australia*	0.70953	0.005
*Potentilla amurensis*	0.13408	0.331

## Data Availability

Raw sequence data generated for this study can be accessed from NCBI PRJNA875579, SRR 21424117 ---- SRR 21424137.

## Supplementary Materials

The following supporting information can be downloaded at: https://www.mdpi.com/article/10.3390/ani14111566/s1. Supplementary Table S1. A detailed description of diet and breeding ecological conditions between two groups; Supplementary Table S2. information of all the fecal samples by 16S rRNA amplicon sequencing; Supplementary Table S3. The full range of plant species from Tianjin Tuanbo Lake; Figure S1: The rarefaction curves between BYG and TBG group based on Sobs index (a) and Shannon index (b); Figure S2: The Venn diagram of BYG and TBG group; Figure S3: The heatmap of microbial structure of all fecal samples at family level.; Figure S4: The heatmap of microbial structure of all fecal samples at class level; Figure S5. ANOSIM results based on the weighted UniFrac distance (a) and unweighted UniFrac distance (b) of all taxa metadata; Figure S6. Microscopy images of plant cell from TBG group; Figure S7. Microscopy images of plant cell from BYG group; Figure S8. The Bubble chart shows the relationship between BYG group and TBG group at phylum/genus level.
