# Supplementary material for: Diet Drives Gut Bacterial Diversity of Wild and Semi-Captive Common Cranes (Grus grus)
Source: Animals (Basel). 2024 May 25;14(11):1566. doi: 10.3390/ani14111566 (PMC11171321; doi:10.3390/ani14111566)
Supplement: Supplementary file 1 [file animals-14-01566-s001.zip › animals-2996203-supplementary/Supplemental materials 2024.doc]

Supplementary information

## 1 Supplementary Tables:

Supplementary Table1. The detailed description of diet and breeding ecological conditions between two groups

| **Group name** | **Type** | **Location** | **Latitude** | **Diet** |
| --- | --- | --- | --- | --- |
| BYG | Semi-captive | Beijing Wildlife Park | 39.496227°N | Fish  *Zea mays*  *Glycine max*  *Phragmites australis*  *Capsella bursa*  *Potentilla chinensis*  *Polygonum criopolitanum*  *Acorus tatarinowii* |
| TBG | Wild | Tianjin  Tuanbo Lake | 38.912993°N | *Triticum aestivum*  *Potentilla chinensis*  *Zea mays*  *Carexbreviculmis*  *Glycine max*  *Phragmites australis*  *Abutilon theophrasti*  *Phragmites australis*  *Artemisia capillaris*  *Ranunculus japonicus* |

Supplementary Table 2. Informations of all the fecal samples by 16S rRNA amplicon sequencing

| Sample/Info | Seq_num | Base_num | Mean_length | Min_length | Max_length |
| --- | --- | --- | --- | --- | --- |
| BYG1 | 76080 | 32336200 | 425.028917 | 225 | 451 |
| BYG2 | 77970 | 32464243 | 416.368385 | 220 | 450 |
| BYG3 | 76421 | 32348833 | 423.297693 | 225 | 451 |
| BYG4 | 80625 | 33970559 | 421.340267 | 260 | 449 |
| BYG5 | 75101 | 31916274 | 424.978016 | 314 | 450 |
| BYG6 | 80956 | 34249373 | 423.061577 | 247 | 449 |
| TBG1 | 82695 | 37590862 | 454.572368 | 50 | 490 |
| TBG2 | 81161 | 37813772 | 465.910622 | 53 | 489 |
| TBG3 | 78330 | 36386805 | 464.532172 | 52 | 490 |
| TBG4 | 76965 | 36147877 | 469.666433 | 291 | 490 |
| TBG5 | 79482 | 36042272 | 453.464583 | 50 | 490 |
| TBG6 | 82333 | 37752758 | 458.537379 | 52 | 487 |
| TBG7 | 81607 | 36202105 | 443.615192 | 50 | 478 |
| TBG8 | 80721 | 37803973 | 468.328849 | 190 | 488 |
| TBG9 | 83401 | 37713878 | 452.19935 | 54 | 486 |
| TBG10 | 84886 | 39868854 | 469.675259 | 120 | 485 |
| TBG11 | 83753 | 39485642 | 471.453464 | 59 | 489 |
| TBG12 | 79160 | 35926640 | 453.848408 | 50 | 490 |
| TBG13 | 76594 | 35556089 | 464.215069 | 50 | 490 |
| TBG14 | 73864 | 33987336 | 460.133976 | 50 | 488 |
| TBG15 | 82506 | 38534724 | 467.053596 | 50 | 489 |

Supplementary Table3.The full range of plant species from Tianjin Tuanbo Lake

| **Family** | **Genus** | **Latin name** | **Distribution location** |
| --- | --- | --- | --- |
| Gramineae | *Echinochloa* | *Echinochloa crus* | island 1 |
|  | *Sorghum* | *Sorghum bicolor* | island6-7 |
|  | *Zea mays* | *Zea mays* | island6-7 |
|  | *Digitaria* | *Digitaria sanguinalis* | island6-7 |
|  | *Phragmites* | *Phragmites australis* | island1,island6-7 |
|  | *Setaria* | *Setaria viridis* | island1,island6-7 |
| Chenopodiaceae | *Suaeda* | *Suaeda glauca* | island1 |
| Composite | *Mulgedium* | *Mulgedium tataricum* | island1 |
|  | *Artemisia* | *Artemisia argyi* | island6-7 |
|  | *Cirsium* | *Cirsium setosum* | island1,island6-7 |
|  | *Sonchus* | *Sonchus wightianus* | island1,island6-7 |
| Labiatae | *Leonurus* | *Leonurus artemisia* | island1 |
| Oleaceae | *Fraxinus* | *Fraxinus chinensis* | island6-7 |
| Salicaceae | *Salix* | *Salix matsudana* | island6-7 |
| Temaricaceae | *Tamarix* | *Tamarix chinensis* | island1,island6-7 |
| Malvaceae | *Abutilon* | *Abutilon theophrasti* | island6-7 |
|  | *Hibiscus* | *Hibiscus trionum* | island6-7 |
| Cucurbitaceae | *Luffa* | *Luffa chylindrica* | island6-7 |
| leguminous | *Sophora* | *Sophora japonica* | island6-7 |
| Asxlepiadaceae | *Cynanchum* | *Cynanchum chinense* | island1,island6-7 |

## 2 Supplementary Figures:

Supplementary Figure1. The rarefaction curves between BYG and TBG group based on Sobs index (a) and Shannon index (b)

Supplementary Figure2. The Venn diagram of BYG and TBG group

Supplementary Figure 3. The heatmap of microbial structure of all fecal samples at family level.

Supplementary Figure4. The heatmap of microbial structure of all fecal samples at class level.

Supplementary Figure5. ANOSIM results based on the weighted unifrac distance (a) and unweighted unifrac distance(b) of all taxa metadata.

Supplementary Figure6. Microspoy images of plant cell from TBG group

Supplementary Figure7. Microspoy images of plant cell from BYG group

Supplementary Figure8. The Bubble chart showing the relationship between BYG group and TBG group at phylum/genus level.
